# Detection of Peanut Allergen by Real-Time PCR: Looking for a Suitable Detection Marker as Affected by Processing

**DOI:** 10.3390/foods10061421

**Published:** 2021-06-18

**Authors:** Africa Sanchiz, Paulina Sánchez-Enciso, Carmen Cuadrado, Rosario Linacero

**Affiliations:** 1 Food Technology Department, SGIT-INIA, Ctra. La Coruña Km. 7.5, 28040 Madrid, Spain; africa.sanchiz@gmail.com; 2 Genetics, Physiology and Microbiology Department, Biology Faculty, Complutense University, 28040 Madrid, Spain; m.paulinasanchezenciso@gmail.com

**Keywords:** real-time PCR, peanut, food allergen, chloroplast marker, DNA isolation

## Abstract

Peanut (*Arachis hypogaea*) contains allergenic proteins, which make it harmful to the sensitised population. The presence of peanut in foods must be indicated on label, to prevent accidental consumption by allergic population. In this work, we use chloroplast markers for specific detection of peanut by real-time PCR (Polymerase Chain Reaction), in order to increase the assay sensitivity. Binary mixtures of raw and processed peanut flour in wheat were performed at concentrations ranging from 100,000 to 0.1 mg/kg. DNA isolation from peanut, mixtures, and other legumes was carried out following three protocols for obtaining genomic and chloroplast-enrich DNA. Quantity and quality of DNA were evaluated, obtaining better results for protocol 2. Specificity and sensitivity of the method has been assayed with specific primers for three chloroplast markers (mat k, rpl16, and trnH-psbA) and Ara h 6 peanut allergen-coding region was selected as nuclear low-copy target and TaqMan probes. Efficiency and linear correlation of calibration curves were within the adequate ranges. Mat k chloroplast marker yielded the most sensitive and efficient detection for peanut. Moreover, detection of mat K in binary mixtures of processed samples was possible for up to 10 mg/kg even after boiling, and autoclave 121 °C 15 min, with acceptable efficiency and linear correlation. Applicability of the method has been assayed in several commercial food products.

## 1. Introduction

Peanut *(Arachis hypogea* L.) is a plant belonging to the Fabaceae family, whose seed fruits are worldwide consumed. A peanut allergy is one of the most common IgE-mediated reactivities to food because of its severity and lifelong persistence [1]. Considerable effort has been spent in characterizing peanut allergens and several allergenic proteins have been identified until now (up to Ara h 18 has been included in the WHO/IUIS Allergen Nomenclature Sub-Committee Database). The major peanut allergens, Ara h 1 (65 kDa, vicilin) and Ara h 2 (17 kDa, conglutin), are recognized by 70–90% of sensitised subjects [2], and Ara h 3 (11 S legumin) has been considered to play a lesser allergenic role [3]. The thermal treatment has a significant effect on peanut immunoreactivity. Roasting peanut enhances its IgE-binding capacity [4], while boiling decreases its allergenicity [5]. According to Cabanillas et al. [6], IgE immunoreactivity of roasted peanut decreased significantly in extreme conditions of autoclaving (2.6 bar, 30 min). Applying DIC treatment (instantaneous controlled pressured-drop, based on 6 bar of pressure for 3 min) to raw and roasted peanut proteins compared to untreated samples resulted in a significant decrease in the protein band of 65 kDa (putative Ara h 1) and the disappearance of immunoreactive bands under 20 kDa [7]. The immunoreactivity changes of peanut proteins following thermal treatment may be due to the modification of protein structure of each individual allergen of peanut as well as their interaction with the food matrix.

Nowadays, as no treatment for food allergies is available, sensitised individuals must avoid the consumption of the offending ingredient, but the presence of allergens in foods can be a consequence of fraudulent substitution or adventitious contamination during food processing at the industrial facilities. Therefore, the development of reliable and specific tools to detect traces of food allergens is indeed essential to improve the quality of life of sensitised individuals, in agreement with the consensus experts [8]. Usually, protein-based assays, such as enzyme-linked immunosorbent assay (ELISA), are used to detect small amounts of proteins from specific foods. DNA-based methodologies, such as real-time PCR (Polymerase Chain Reaction) and genosensors, have been proposed as specific, sensitive, and reliable alternatives to ELISA since DNA molecules preserve their integrity better than proteins [9]. Protein methods based on mass spectrometry approaches have also been developed and applied for peanut allergen analysis in different food product categories [10]. Up to now, several methods have been performed for peanut allergen detection, either relying on protein or DNA-analysis [11,12,13,14,15,16]. Foods, including peanuts, are usually thermally treated to preserve food safety or even modify allergenic reactivity [17] but maintaining or improving organoleptic and functional properties [18]. The protein solubility can be highly affected by thermal food processing, and subsequent detection with protein-based techniques might be hampered [19]. In contrast to protein-based techniques, DNA-based assays have been proposed as a reliable, sensitive, and specific alternatives for food allergen identification. Although DNA is a very stable molecule, fragmentation and/or degradation of DNA molecules after severe treatments have been reported by several authors [20,21]. Some studies have analyzed the effect of many different treatments (boiling, high hydrostatic pressure (HHP), autoclave, frying, roasting) on the detection of different DNA targets in peanut, hazelnut, walnut, almond, or pistachio, among others [22,23,24,25,26].

DNA-based methods, mainly relying on real-time PCR, have been widely applied to peanut detection in foods, as reviewed by Zhan et al. [27]. Specificity, sensitivity, and potential of quantification of a real-time PCR method for allergen detection can be compromised by the selected target sequence. Either multi-copy (such as ITS or chloroplast sequences or genes) or single/low-copy genes have been used as targets for real-time PCR based detection methods for nut analysis [9,15,27,28,29,30,31,32]. Most of the real-time PCR methods for peanut detection used the Ara h 2 coding sequence as the target gene reaching sensitivity levels around 2–10 ppm of peanut in food products [15,16]. When the ITS region is used as a target, the sensitivity is increased (0.1 ppm) [15]. The data with chloroplast sequences, such as atp 6 or mat k, indicate that these sequences are powerful markers for the quantitative detection of trace amounts of peanut in commercial food products [30,31,32]. The objective of this study was comparing the performance of chloroplast (mat k, trnH-psbA and rpl16) and nuclear (Ara h 6 allergen-coding gene) marker sequences for peanut detection by real-time PCR. The validation of the set-up real-time PCR method with the selected marker is also intended. Additionally, we aimed to analyze the effect of thermal food processing (boiling, autoclaving, and DIC) on peanut detection in complex food products by real-time PCR.

## 2. Materials and Methods

### 2.1. Plant Material and Treatments

Peanuts *(Arachis hypogaea*) were provided by Productos Manzanares S.L. (Cuenca, Spain). Several thermal and pressure-based treatments were performed on whole peanuts. One hundred grams of peanuts, immersed in distilled water, were boiled for 60 min or autoclaved (Compact 40 Benchtop, Priorclave, London, UK) at 121 °C (1.20 bar) and at 138 °C (2.56 bar) for 15 and 30 min, as previously described [33]. Peanuts were also subjected to controlled Instantaneous Depressurization (DIC) treatment, performed at the La Rochelle University (LaSiE). DIC treatment was carried out following a factorial experimental design previously described [34]. In this experiment, the moistened whole nuts are placed in a processing chamber and exposed to steam pressure (7 bar) at high temperature (up to 170 °C), over a short time (120 s). An instant pressure drop towards a vacuum at about 50 mbar follows this high-temperature-short time stage. This abrupt pressure drop, at a rate ΔP/Δt higher than 5 bar/s, simultaneously provokes an auto-vaporization of a part of the water in the product, and an instantaneous cooling of the products, which stops thermal degradation. After treatments, peanuts were freeze-dried (Telstar Cryodos, Terrasa, Spain), defatted using n-hexane (34 mL/g) for 4 h. Flours were passed through a 1 mm mill and stored at 4 °C until use.

### 2.2. Binary Mixtures

Binary mixtures (spiked samples) of untreated (control) and treated (boiled, B60; autoclave 121 °C at 15 min (A1) and at 30 min (A2); autoclave 138 °C at 15 min (A3) and 30 min (A4) and DIC (DIC) defatted flours were performed in spelt wheat as described elsewhere [26]. Thus, mixtures containing 10, 100, 1000, 10,000, and to 100,000 mg/kg (0.01–10%) of peanut in wheat were prepared in a final weight of 25 g. Spiked samples were mixed using a kitchen robot (Thermomix 31-1, Vorwerk Elektrowerke, GmbH & Co. KG, Wüppertal, Germany). The mixture containing 10% of each peanut sample (100,000 mg/kg) was prepared by adding 2.5 g of the nut flour to 22.5 g of spelt wheat flour, and followed by 10-fold dilutions, homogenizing with the kitchen robot (Table S1).

### 2.3. DNA Isolation and Conventional PCR

Isolation of DNA was performed following 3 different protocols.

Protocol 1. Genomic DNA was obtained using DNeasy Plant Pro kit (Qiagen, Hilden, Germany), with slight modifications. Briefly, 80 mg of binary mixtures and other plant species were homogenized in 1 mL of CD1 buffer with 5 µL of 25 mg/mL RNAse using Tissue Lyser (TissueLyser II, Qiagen, Hilden, Germany) for cycles of 2 min at 24 1/s of frequency with 30 s of pause. After centrifugation for 5 min at 11,000 rpm, 500 µL of supernatant were collected and 250 µL of CD2 buffer were added. Incubation for 20 min in ice was included after this step, followed by centrifugation at 11,300 rpm for 2 min. Five hundred µL of supernatant were mixed with 1 volume of APP buffer before loading the column. Two steps of washing with AW2 buffer were performed, and DNA was eluted in 50 µL of pre-warmed deionized water (protocol 1). With the aim of performing a representative (compared to genomic DNA kit) but chloroplast-focused, faster and/or cheaper DNA isolation protocols, DNA from the same quantity of flour was isolated with protocols 2 and 3.

Protocol 2. DNA was obtained using SpeedTools plasmid DNA purification Kit (Biotools, Loganholme, Australia) following the manufacturer instructions. In addition, 80 mg of peanut and binary mixtures were homogenized in 350 µL of resuspension buffer (including 0.5 mg/mL of RNase) using a tissue lyzer at the same conditions described above. After that, 350 µL of lysis buffer were added and tubes were mixed by inversion; after the addition of other 400 µL of the neutralization buffer, samples were centrifuged for 5 min at 13,000 rpm, the supernatant was collected and loaded to the column. Two steps of washing were performed, and DNA was eluted in 80 µL of pre-warmed deionized water.

Protocol 3. In addition, 80 mg of peanut or binary mixture’s flours were homogenized in 700 µL of solution I (glucose 50 mM, Tris HCl 25 mM, EDTA 10 mM) using tissue lyzer at the same conditions described above. Centrifugation at 3000 rpm for 5 min was performed, and 500 µL of supernatant was collected and centrifuged at 12,000 rpm for 10 min. The pellet was suspended in 500 µL of MLB (NaCl 150 mM, Na_2_EDTA 50 mM, Tris HCl 10 mM), 250 µL of solution II (2% SDS, NaOH 0.4 M) and 500 µL of solution III (29.5% acetate, pH 4.8), and centrifuged at 13,000 rpm for 5 min at room temperature. Eight hundred µL of supernatant was kindly mixed with 700 µL with cold isopropanol, and centrifuged 15 min at 13,000 rpm. The pellet was washed with 500 µL of cold ethanol, centrifuged 5 min at 13,000 rpm, and completely dried before suspended in 50 µL of pre-warmed deionized water. 

DNA from food products was isolated using NucleoSpin kit (Macherey-Nagel, Düren, Germany) following the manufacturer instructions with minor modifications [35]. Quality and quantity of isolated DNA was analyzed by spectrophotometry using NanoDrop™ One (Thermo-Fisher, Waltham, MA, USA) and electrophoresis by 0.8% agarose-gels. 

End-point PCR using universal eukaryotic primers targeting the nuclear 18S rRNA gene were performed as described by Sanchiz et al. [36] (Table 1). These reactions were carried out in 20 µL, containing 25 ng of DNA, 250 nM of each primer and 1XFastStar PCR Master Mix (Biotools, Loganholme, Australia). SensoQuest LabCycler (Progen Scientific Ltd., London, UK) was programmed with an initial denaturation step at 95 °C, 5 min, followed by 35 cycles of denaturation at 94 °C for 1 min, annealing at 60 °C for 30 s and elongation at 72 °C for 45 s, and a last step at 72 °C for 5 min.

### 2.4. Primers, Probes, and Sequencing

Three chloroplast markers from *Arachis hypogaea* were selected based on previous literature [30], named mat K, trnH-psbA, and rpl16 (KJ468094.1). A slight modification on one of the primer sequences for a mat K target was done, due to amplification performance reasons. Moreover, Ara h 6 allergen-coding region of peanut (AF092846) was selected as a nuclear low-copy target, and specific primers and a Taqman probe were designed using primer3 software. In silico analysis of these sequences was also performed using BLASTn from NCBI, searching for homologue sequences from other species, especially legumes and tree nuts. Moreover, end-point PCR was performed with peanut and other species DNA using specific primers (Table S2), following the same program that was described in Section 2.4. Then, Sanger sequencing of partial rpl16, trnH-psbA, and Ara h 6 sequences was performed in an ABI PRISM 3700 sequencer (Applied Biosystems, Foster City, CA, USA) from the Genomics Service (Universidad Complutense de Madrid, Spain). In the case of Ara h 6, resulted amplicons were cloned into the pCR™4-TOPO^®^ Vector using TOPO^®^ TA Cloning^®^ Kit (Invitrogen, Inc., Paisley, UK) following the manufacturer’s instructions. Sequences were analyzed using Bioedit Software (Ibis Biosciences, Carlsbad, CA, USA). Final sequences of primers and probes, their final concentration used in real-time PCR reactions, and amplicon sizes are included in Table 1.

### 2.5. Real-Time PCR

Real-time PCR assays were performed with 7900HT Fast real-time PCR (Applied Biosystems, CA, USA), in a volume of 20 µL containing 5 µL of DNA at different concentrations, different final concentration of primers and probes (Table 1), and 10 µL of TaqMan ^®^ Gene Expression Master Mix (Applied Bio-system, CA, USA). The used program included an initial denaturation at 95 °C for 10 min followed by 40 cycles of denaturation at 95 °C for 15 s and primer annealing and elongation at 60 °C for 1 min.

The cycle threshold (Ct) value, obtained from 10-fold serial dilutions of peanut DNA in deionized water and different points of binary mixtures containing from 100,000 to 0.1 mg of peanut per kg of mixture, was used to generate standard curves for real-time PCR. The efficiency (10^(−1/slope)^−1) of each reaction was calculated from the slope of each standard curve (Ct vs. log DNA content or Ct vs. log Quantity of peanut nut flour). Sensitivity and limit of detection (LOD) were determined taking into account the lowest amount of target that can be detected in at least 95% of the cases [37]. Specificity of primers was tested by real-time PCR, by means of the amplification of 10 ng of isolated DNA from different species.

### 2.6. Statistical Analysis 

The significance of differences (*p* < 0.05) between the Ct values of each spiked level for each treatment (boiling, DIC, and autoclave processing) compared to untreated control was evaluated by a Student’s *t*-test using the GraphPad Prism Program. 

## 3. Results and Discussion

### 3.1. Selection of the Suitable Target

Four target regions were selected for specific detection of peanut: three chloroplast markers, rpl16, trnH-psbA, and mat K and a nuclear allergen-coding gene, Ara h 6. As mentioned in the Materials and Methods section, we first reviewed the available literature in DNA-based methods for peanut allergen detection, selecting rpl16, trnH-psbA, and mat K because of their better results regarding sensitivity, efficiency of the amplification, and specificity [30]. Puente-Lelievre et al. designed a multiplex assay, based on chloroplast markers, to detect allergenic peanut. Multicopy sequences, such as chloroplast or ITS sequences, have been proposed as very sensitive markers for trace food allergen detection, not only in peanut but in other nuts [15,31,32,33,34,35,36,37,38,39,40,41,42]. Low/single copy number genes have also provided specific and sensitive results in real-time PCR [9,15]. Thus, we designed primers and a Taqman probe for the amplification of the allergen-coding gene Ara h 6, which is a major allergen in peanut encoding for a 2S albumin, aimed at detecting peanut traces in food.

**Figure 1:**
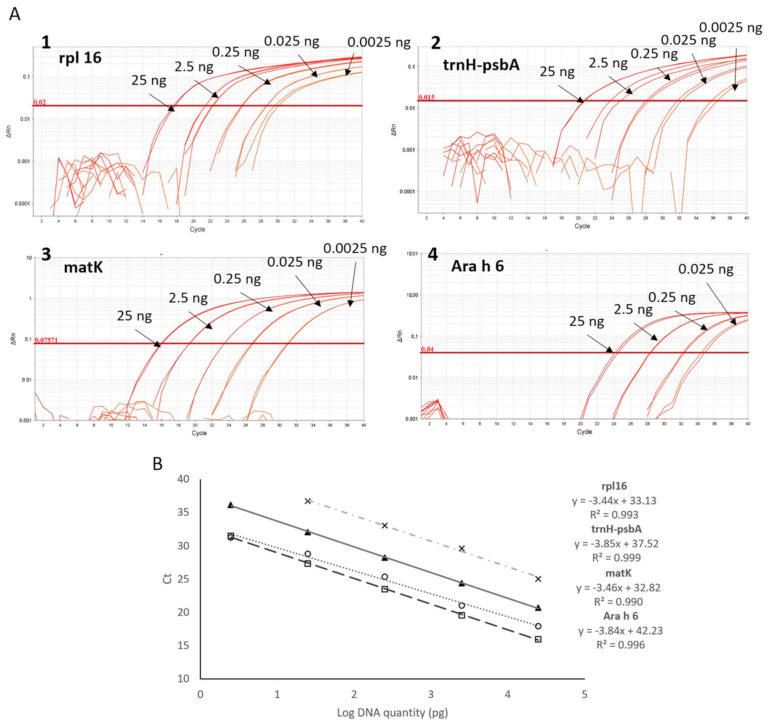
Calibration curves for the four selected targets. (**A**) Representative amplification plots of calibration curves of rpl 16 (1), trnH-psbA (2), mat K (3), and Ara h 6 (4), obtained by real-time PCR amplification of serial diluted peanut DNA (from 25 ng to 0.0025 ng); (**B**) calibration curves of the four targets (“cross” for Ara h 6; “circle” for rpl 16; “triangle” for trnH-psbA and “square” format (K) represented by plotting Ct against the logarithm of peanut DNA quantity. Slope and correlation coefficient (R2) for each target are shown.

Amplification plots, calibration curves with serial diluted DNA, and binary mixtures spiked with peanut of the four targets are showed in Figure 1 and Table 2, respectively. All DNA extracts used in these experiments was isolated with protocol 1, described in the Materials and Methods section.

Regarding the rpl16 target, 95% of efficiency of the amplification reaction and 0.993 of correlation coefficient was obtained (Figure 1). However, calibration curves with binary mixtures using same primers and probe did not show acceptable efficiency (113.52%) or a correlation coefficient (0.970) (Table 2).

The trnH-psbA marker showed lower efficiency values than expected (84%), with an adequate correlation coefficient (0.9912) with serial dilutions of peanut DNA (Figure 1). When curves were performed with binary mixtures containing known amounts of peanut in wheat, efficiency and R^2^ were within the adequate ranges. Regarding sensitivity, at least 1 mg/kg of peanut in binary mixtures was detected in 100% of the replica (Table 2).

The mat K primers/probe yielded good results regarding amplification efficiency, after analyzing both performed calibration curves, 91.36% for serial diluted DNA, and 108.30% in binary mixtures curves. A linear dynamic range was obtained between 25 and 0.0025 ng with an R^2^ coefficient of 0.994 in the 10-fold diluted DNA curve (Figure 1). After representing the mean Ct value and the log of the quantity of peanut flour in the mixtures, R^2^ was 0.995, in the linear range between 100,000 and 1 mg/kg (Table 2).

Ara h 6 primers and probe were designed based on the sequencing information of some clones (Figure S1). Calibration curves using primers for peanut Ara h 6 coding gene (Figure 1 and Table 2) did not show acceptable efficiency (within the range of 90–110%), although good linearity was obtained in both cases (R^2^ > 0.990). Twenty-five nanograms of peanut DNA (first point of the DNA curve) were detected after 25 cycles of amplification, almost eight cycles after the same sample assayed with primers targeting chloroplast markers as mat K and rlp16.

Regarding the specificity of chloroplast targets, trnH-psbA, and mat K markers, the results after assaying 10 ng of DNA from several species by real-time PCR are presented in Table 3. The mat K primers and probe specificity showed Ct ranged between 34.44 ± 0.27 for pistachio and 39.70 ± 0.42 for apple (Table 3). This amplification was not considered significant since 10 ng of peanut DNA would be detected at cycle 18 (by DNA curve extrapolation), being even more than 15 cycles earlier than those species. However, the real-time PCR assay with rpl16 primers/probe showed reactivity with some legumes, beyond peanut (chickpea, soybean, lentil or beans). Partial sequencing of the rpl16 gene, using specific primers (Table S2), revealed that the rpl16 sequence from other legumes were identical to the rpl16 peanut including the primers and probe position (data not shown). Therefore, the rpl16-based protocol was not specific enough for peanut detection, using the same plant species and PCR conditions previously reported [30], being discarded. On the other hand, when DNA from non-target plant species was analyzed with Ara h 6 gene primers/probe, positive signals were obtained at Ct < 35, and putative LOD was too high (1000 mg/kg), thus discarding this marker for further peanut analysis.

### 3.2. Effect of Thermal Treatments on Peanut Detection

**Figure 2:**
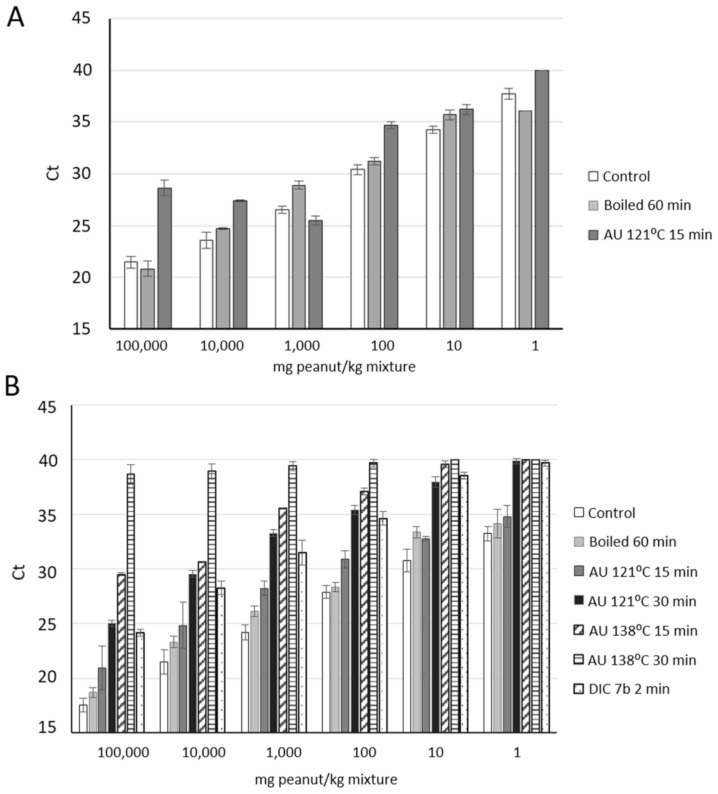
Real-time PCR detection of peanut targeting trnH-psbA (**A**) and mat K (**B**) chloroplast marker in untreated and treated samples. Binary mixtures were performed with known amounts of untreated (control, white columns), boiled for 60 min (grey) or autoclaved at 121 °C (1.2b) for 15 min (dark grey) peanut in wheat (from 100,000 to 1 kg/kg) for the analysis using both targets. Other three autoclave conditions (columns in black, diagonal, and horizontal stripes) and DIC processing (dotted column) were evaluated by real-time PCR targeting mat K sequence. Mean Ct values against mg of untreated or treated peanut by kg of mixture are represented.

In the food industry, processing is frequently applied in order to improve safety and organoleptic properties. In the recent years, many researchers have demonstrated that allergen detection is importantly affected by food processing, not only in protein-based detection methodologies but also in DNA-based ones [9,43], being extensively reviewed. It has been defended that the food-processing effect on DNA target detection by PCR should be always considered, although DNA is a very stable molecule, especially compared to proteins. It has been possible to detect DNA markers even in harsh thermal conditions, including autoclaving, roasting, boiling, etc. [26,44]. In order to establish the real influence of any processing, many authors have supported the necessity of analyzing the same kind of model mixtures in control and treated samples [45]. For this reason, binary mixtures were also made with treated flours in the same wheat matrix. Here, we described the effect of several treatments, based on temperature (boiling) or heat combined with pressure (Depressurized Instant Controlled, named DIC and autoclaving at different conditions) on the detection of specific peanut chloroplast DNA markers. A calibration curve has then been performed for each treatment, plotting the average Ct values against the log of the quantity of peanut flour in mixture. NTC (non-template control) samples were not always detected (N.D.), and DNA was first isolated with standard protocol 1. Regarding the detection of the trnH-psbA marker, a PCR efficiency of 106% and coefficient of correlation of 0.970 in curves set up from boiled mixtures, and amplification was delayed two cycles compared to untreated control (Figure 2, light grey columns). However, when pressure and temperature were applied, in the first autoclave condition (at 121 °C for 15 min), the detection of trnH-psbA was difficult. Linear range was not obtained, and Ct > 29 was obtained in samples with 10% *w*/*w* of treated peanut (or 100,000 mg/kg) in the mixture, compared to Ct ~ 20 when peanut was kept untreated (Figure 2A).

The effect of autoclaving for longer periods of time was also analyzed, but the tendency was similar to that obtained in softer conditions of autoclave (data not shown). Amplicon size of trnH-psbA was small, just 68 pb, and this fact usually contributes to better results regarding sensitivity when DNA integrity is affected. Recently, we designed a real-time PCR assay to detect the cashew presence in food, it being possible even in autoclaved samples at 138 °C for 15 min, maintaining linearity of the curves up to 1000 mg/kg [36]. In that case, amplicon size was only 65 pb, targeting a partial Ana o 1 allergen coding gene. Nevertheless, amplicon size is not the only important factor influencing the capacity to detect the target in samples with compromised DNA integrity. From our point of view, it becomes essential to analyze, experimentally, the influence of several common food technological treatments on the detection capacity of any specific target. Identically, the approach targeting for the mat K was assayed, showing suitable detection from boiled peanut mixtures for 1 h and comparable performance to untreated mixtures (R^2^ > 0.98 and PCR of 106.7% efficiency). Efficiency and correlation coefficient of the curves were within acceptable ranges when binary mixtures were performed with other treated peanut flour as DIC and autoclave at 120 °C for 15 and 30 min, although detection was significantly delayed several cycles compared to control mixture (Table S3).

According to our results, the influence of DIC treatment, based on high temperature (up to 180 °C) and pressure (7b) for a very short time (2 min), on mat K target detectability was similar to the observed effect of autoclave at 121 °C for 30 min, with no significant differences among the Ct values in different spiked levels (*p* > 0.05). In both cases, it was possible to detect up to 1 mg/kg of peanut but in less than 50% of the replica (Table S3). Our group recently described for the first time the effect of this novel thermal treatment on real-time PCR detection of three tree nuts allergen-coding sequences [34]: Cor a 9 from hazelnut, Pis v 1 from pistachio, and Ana o 1 from cashew. In that article, we reported the capacity to detect and quantify DIC-treated samples in mixtures when the allergenic ingredient was around 100,000 and 1000 mg/kg. Here, the detection system would allow detecting and even quantifying the presence of treated peanut when it is around 100,000 and 100 mg/kg in a mixture. When autoclave at 138 °C was applied on peanut and DNA from the mixtures was obtained, linearity of the curves (representing mean Ct of several spiked level vs. log quantity of peanut in each mixture) was not maintained, and efficiency was slightly higher than the acceptable 110% (Table S3). Detection was not possible in AU 138 °C 30 min samples, with Ct > 38 in all the spiked levels.

It resulted in being interesting to observe that maximum obtained Ct value was higher in samples containing treated peanut comparted to those with untreated nuts. Thereby, as an example, in samples with 1 mg/kg (LOD of the system) of untreated/control peanut, Ct was ~33, and then Ct values were not in the calibration curve. However, for mixtures containing boiled peanut, the same peanut quantity was detected at cycle 36, within the linear dynamic range of the calibration curve (Figure 2 and Table S3). A similar effect can be discussed when we analyzed the results from our reference [30]. Authors prepared several food matrices spiked with known amounts of peanut flour (muffins, cookies, sauces, etc.) and generated calibration curves by plotting Ct values against the log of peanut concentration. Obtained Ct values for the lowest spiked level were different depending on the matrix (Ct 32–38), and good linearity and efficiency were obtained in some of them. Thus, thermal treatment is contributing to the fragmentation or degradation of DNA molecules affecting the final Ct values.

As mentioned, real-time PCR methods for peanut detection have been performed before targeting different sequences, but only a few analyzed the effect of processing on DNA detectability, compared to untreated samples [15,16,39]. Among them, usually peanut-containing foodstuff that requires some kind of thermal treatment, such as cookies, sauces or doughs, is prepared and analyzed. Here, we incorporated peanut samples processed by a plethora of conditions regarding temperature, pressure and time, observing the influence on the target detectability.

### 3.3. Comparative Analysis of DNA Isolation Methods

To compare DNA isolations methods, we decided to perform the real-time PCR assay targeting mat K chloroplast gene, based on the primers and probe published by Puente-Lelievre et al. [30], with a slight modification on the reverse primer to improve reaction performance. Mat K primers/probe yielded good results with DNA obtained with protocol 1, as presented in Section 3.1. Thus, absolute LOD was established at 0.0025 ng (2.5 pg) of peanut DNA and the relative LOD, calculated with standard curves based on binary mixtures spiked with known quantity of peanut, was established at 1 mg of peanut per kg of mixture, with Ct max of 33.22 ± 0.20 (Figure 1 and Table 2) [31]. Since the lowest amplified level is within the linear range of the standard curve, 1 mg/kg is also considered as the limit of quantification (LOQ). The detection below 1 mg/kg was possible, but Ct was not within the calibration curve (Table 3). Therefore, the obtained results of absolute LOD and PCR efficiency were more promising than those recently reported by Puente-Lelievre et al. [30]. In the particular case of mat K, when it was analyzed as a single marker, authors obtained standard curves with a correlation coefficient of 0.98 and PCR efficiency of 88%, calculated from the curve slope. With the slight modification on the reverse primer published by these authors, we acquired R^2^ and efficiency within the acceptable ranges for this single chloroplast marker. LOD for mat K target was 1 mg/kg in both previously published study and ours, using different food matrices.

**Figure 3:**
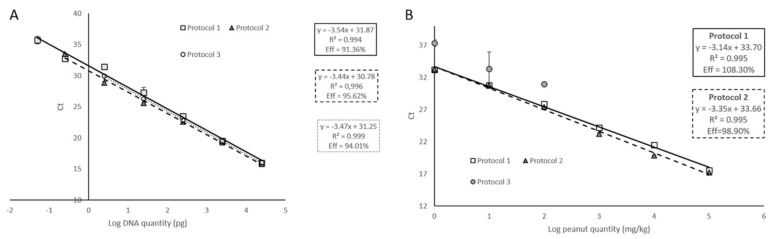
mat K target detection analyzing peanut DNA samples isolated with three different protocols. (**A**) Calibration curves of real-time PCR assay using mat K specific primers and probe, performed with 10-fold serial diluted peanut DNA isolated by three different isolation protocols (1–3); (**B**) calibration curves performed with DNA from binary mixtures of peanut in wheat, using DNA obtained with the three different protocols. Efficiency and R^2^ values are included for the two kit-based DNA isolation protocols. Only Ct values obtained after DNA isolation by protocol 3 are shown as grey circles for comparison purposes (samples containing 100, 10, and 1 mg of peanut/kg of mixture). Mean and standard error are represented.

Protocols 2 and 3 were planned to be faster and cheaper methods to obtain quality DNA from peanut samples, and more focused on the isolation of chloroplast DNA, compared to standard genomic DNA extraction protocol (protocol 1). Thus, detection of the chloroplast marker, mat K, might be facilitated. Moreover, we aimed to use this isolated DNA for a subsequent future use in a specific peanut genosensor, which requires a rapid, reproducible, and economically worthwhile DNA isolation step. General results about the reaction performance using DNA isolated with protocols 2 and 3 are shown in Figure 2. Both calibration curves from 10-fold diluted peanut DNA showed good linearity with correlation coefficients higher than 0.99 and efficiencies of the amplification within the acceptable ranges (90–110%), not significantly different to the performance observed when protocol 1 was applied (*p* > 0.05; Figure 3A). Similar results were obtained when calibration curves were performed using binary mixtures spiked with known amounts of untreated peanut flour, with DNA obtained by means of protocol 2 (Figure 3B, triangle). In contrast, DNA from these samples obtained with protocol 3 or in-house protocol did not allow detecting peanut with enough reproducibility and feasibility, since, with binary mixtures, each point is obtained from an independent DNA isolation and is not built by serial dilution of the DNA (Figure 3B).

**Figure 4:**
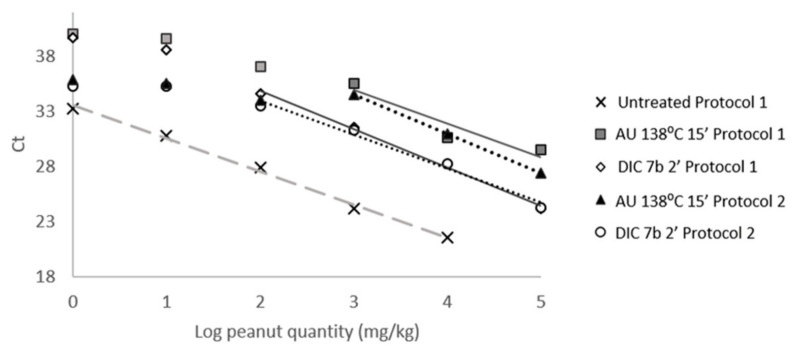
Influence of the DNA isolation method in the performance of mat K detection in processed matrices. Amplification of mat K was analyzed in two treated peanut samples, by AU 138 °C 15 min and DIC 7b 2 min, which DNA was isolated by protocols 1 and 2. Curves were performed by plotting mean Ct and log of the peanut quantity. As a reference, a curve performed with untreated peanut in wheat is included, using protocol 1 for DNA isolation (cross).

As described before with untreated samples, DNA from several points of binary mixtures prepared with processed peanut flour (DIC 7b 120 s and AU 138 °C 15 min) was isolated using protocols 2 and 3, and compared to standard protocol 1 (Figure 4). Standard curves built with DNA from mixtures containing from 100,000 mg/kg to 1 mg/kg of DIC-treated peanut, obtained from protocols 1 and 2, showed a linear dynamic range extended 4 log_10_, up to 100 mg/kg of treated with R^2^ > 0.99 (Figure 4). In the case of autoclave-treated spiked samples, DNA obtained with protocol 2 allowed for building a curve with a better correlation coefficient than the one with protocol 1 (R^2^ 0.998 vs. 0.885). With DNA obtained with protocol 3, nevertheless, it was not possible to achieve consistent and reliable data, it being complicated to obtain enough DNA from such processed samples (data not shown). With all these data, it can be concluded that protocol 2 allows the peanut DNA isolation in a cheaper and faster manner than standard genomic DNA isolation protocol (here protocol 1), even in processed matrices. Performance of the reactions is not affected, although it is not improving chloroplast marker detection compared to the standard protocol. DNA from other species, isolated with this protocol, should be analyzed by real-time PCR; thus, this protocol might be established as the routine DNA isolation method in the future, for sample analysis by a specific genosensor for peanut detection.

### 3.4. Applicability of the Peanut Mat K-Based Detection Assay

Finally, we have confirmed the possible applicability of the real-time PCR assay, based on mat K target amplification, for the detection of peanut traces in foodstuff. All food samples were amplified targeting the 18S rRNA gene as endogenous control [36], confirming the presence of isolated DNA and the absence of putative co-isolated PCR inhibitors together with the DNA. A 2-fold dilution of the isolated DNA was used, and conventional end-point PCR using universal eukaryotic primers [36] was performed with DNA from food before specific peanut detection. We analyzed thirteen commercial food samples by mat K based real-time PCR assay and results are shown in Table 4. One of them is the cereal bar I, which according to the label contents around 35% of peanut, together with hazelnut, obtaining a mean Ct near to 18. Four of them showed Ct values higher than 36, which might be considered as negative results regarding peanut detection. Cereal bar III declared the presence of peanut/almond traces and 10% of hazelnut content, resulting in a mean Ct of 33. Possible peanut contamination should be considered in food named Chocolate bar I, whose label indicates tree nut traces. Interestingly, 3 out of 13 foods, named chocolate bar II, cookies with fiber and cookies with chocolate, did not declare presence of any allergen on their label; however, Ct 31–32 were obtained and possible contamination should not be discarded. We could estimate the present peanut content in mg/kg by substitution in the standard curve (Table 2). These Ct values are high, are included in the linear range of the standard curve, and those foods might be containing between 10 and 1 mg/kg of peanut (Table 4), or even more if DNA has been damaged during processing, as described above. Comparison with commercial ELISA tests would be interesting in order to determine the feasibility of this assay.

## 4. Conclusions

In this study, a real-time PCR assay addressed to detect peanut in complex food samples has been performed. A general workflow regarding protocols, targets, and main findings is represented in Figure S2. Several gene targets and DNA isolation methods have been proposed and compared in this work. As markers, three chloroplast markers (trnH-psbA, rpl16, and mat K) and one nuclear marker (Ara h 6 allergen coding sequence) were analyzed regarding sensitivity, efficiency, and specificity of each real-time PCR method. The experiments based on trnH-psbA or mat K target detection were the most specific, reliable and sensitive enough for the detection of peanut. We have improved the reaction efficiency of the single mat K reaction by a slight modification on the primer sequence, compared to the available literature. Moreover, different conditions of heat, pressure, and time (as boiling, autoclaving, and DIC. processing) were applied to peanut, and their influence on the amplification of trnH-psbA and mat K targets has been determined. According to our results, the mat K-based real-time PCR method is suitable for reliable for reliable detection of peanut in processed samples, even after application of a plethora of thermal and pressure-based treatments. Detection of mat K in binary mixtures of processed samples was possible up to 10 mg/kg even after boiling, and autoclave 1.2 bar 15 min, with acceptable efficiency and linear correlation. Applicability of the method has been assayed in several commercial food products. DNA isolation kits based on silica membranes resulted in being more adequate for the obtaining of quality DNA from complex food matrices, named in this work protocol 1 for the isolation of total DNA and 2 for the obtainment of plasmid-enrich DNA. These two protocols have been compared, showing the same amplification performance for mat K target. Protocol 2 resulted in a cheap and fast methodology that might be applied for the DNA isolation step in the future, in the development of novel and innovative detection systems.

## Figures and Tables

**Table 1:** DNA sequences of primers and probes.

Oligonucleotide	Sequence (5′ → 3′)	Amplicon (bp)	Reference
mat K fw	TGGACTCGCCTCTGGTCAT	104	
mat K rv	CTGCATATCCGCAAATACCG		[30] *
mat K probe	FAM-CATCCCATTAGTAAGCCCGTTTG-BHQ		
trnH-psbA fw	AGGAGCAATAGAAACTGCGT	68	
trnH-psbA rv	TTTTTGTCTTAAGGGATACGAGT		[30]
trnH-psbA probe	6FAM-TGATATTGCTCCTTTACTTTCAAAA-BHQ1		
rpl16 fw	GCGATGGGAACGACGAAAAC	69	[30]
rpl16 rv	TTAGTTCGTTCCGCCATCCC		
rpl16 probe	6FAM-ACCTAAGATTCATTTGACGGGA-BHQ1		
Ara h 6 fw	AGTGCGATAGGTTGCAGGAC	107	
Ara h 6 rv	AAATCGCAACGCTGTGGTG		This article
Ara h 6 probe	6FAM-GCAAATGGTGCAGCAGTTCAAGAG-BHQ1		
Universal 18S fw	CGCGAGAAGTCCACTAAACC	64	[36]
Universal 18S rv	CCTACGGAAACCTTGTTACGA		

* Based on that article with slight sequence modification.

**Table 2:** Comparative data of calibration curves from binary mixtures of peanut in wheat, analyzed by real-time PCR using specific primers and probe for the four selected targets. Obtained Ct mean and SD for each spiked level is shown, as well as calibration curves’ parameters (slope, coefficient correlation, and efficiency of the reaction). All DNA samples were isolated by protocol 1.

Peanut Quantity (mg/kg)	rpl 16	trnH-psbA	mat K	Ara h 6
100,000	15.54 ± 0.02	21.45 ± 0.56	17.55 ± 0.17	25.97 ± 0.02
10,000	18.98 ± 0.07	23.55 ± 0.77	21.52 ± 0.30	28.71 ± 0.11
1000	22.10 ± 0.22	26.51 ± 0.33	24.17 ± 0.17	31.78 ± 0.22
100	26.55 ± 0.02	30.39 ± 0.46	27.89 ± 0.14	34.72 ± 0.05
10	27.24 ± 0.01	34.26 ± 0.36	30.77 ± 0.25	36.65 ± 0.19
1	29.35 ± 0.02	37.70 ± 0.17	33.22 ± 0.20	37.61 ± 1.35 *
0.5	-	-	32.74 ± 0.15 *	-
0.1	-	-	33.87 ± 0.58 *	-
Slope	−3.04	−3.35	−3.14	−2.74
Efficiency (%)	113.52	98.81	108.30	131.95
R2	0.970	0.991	0.995	0.994

* Detection is possible, but Ct is not considered in the calculation of the parameters of the calibration curve.

**Table 3:** Specificity of the three selected targets. In addition, 10 ng of DNA from different plant species was assayed at least in triplicate. Protocol 1 was used to obtain genomic DNA. N.D. not detected. “-not assayed”.

Common Name	Scientific Name	Ct ± SD			
		rpl16	trnH-psbA	mat K	Ara h6
Apple	*Malus domestica*	-	-	39.70 ± 0.42	-
Lentil	*Lens culinaris*	28.30 ± 1.00	N.D.	39.51 ± 0.68	-
Lemon	*Citrus x limon*	-	-	37.24 ± 0.21	-
Lupin	*Lupinus albus*	29.10± 0.25	-	37.71 ± 1.22	34.60 ± 0.28
Almond	*Prunus dulcis*	-	N.D.	37.79 ± 1.24	-
Green bean	*Phaseolus vulgaris*	38.7 ± 0.14	N.D.	38.25 ± 0.79	-
Kiwi	*Actinia deliciosa*	-	-	N.D.	-
Carob	*Ceratonia siliqua*	28.54 ± 0.46	-	35.46 ± 2.48	-
Walnut	*Juglans regia*	-	N.D.	35.55 ± 0.26	-
Pear	*Pyrus pyrifolia*	-	-	N.D.	-
Fababean	*Vicia faba*	28.44 ± 0.70	N.D.	36.20 ± 1.62	-
Chickpea	*Cicer arietinum*	26.83 ± 1.57	N.D.	37.73 ± 2.60	31.62 ± 0.01
Soy	*Glycine max*	29.44 ± 0.03	N.D.	39.06 ± 1.11	-
Pistachio	*Pistacia vera*	-	35.33 ± 0.94	34.44 ± 0.27	34.90± 0.08
Hazelnut	*Corylus avellana*	-	36.97 ± 0.38	37.51 ± 0.48	34.22 ± 0.01
Cashew	*Anacardium occidentale*	-	37.39 ± 1.48	37.39 ± 1.48	-
Grass pea	*Lathyrus sativus*	37.08 ± 0.83	-	37.77 ± 0.89	-
Chestnut	*Castanea sativa*	-	-	N.D.	-
Pea	*Pisum sativum*	N.D.	N.D.	38.47 ± 0.88	33.74 ± 0.14
Wheat	*Triticum spelta*	N.D.	35.70 ± 0.30	N.D.	35.17 ± 0.43

**Table 4:** Detection of mat K target in several commercial food products by real-time PCR. Mean Ct and standard deviation is shown. Measurements of the same sample were performed at least twice in two different DNA extractions. N.D. means that non-signal after 40 cycles of amplification. All samples were first assayed for amplification inhibitor presence using eukaryotic universal primers.

Food	Food Allergen Declaration	Ct ± SD	Peanut mg/kg *
Cereal Bar I	Peanut (35%), Hazelnut (24%)	17.97 ± 0.26	>10^5^
Cereal Muesli	Tree nut and peanut traces	38.73 ± 0.76	<LOQ
Cereal Bar II	Almond and tree nuts	32.40 ± 0.29	2.5
Cereal Bar III	Hazelnut (10%), Almond and Peanut traces	33.74 ± 0.29	<1
Cereal Bar IV	May content tree nut traces	35.90 ± 0.94	<LOQ
Chocolate with pistachio	Pistachio (5%), Almond, Hazelnut, tree nut traces	36.80 ± 0.43	<LOQ
Vegetal Burger	May content tree nut traces	39.17 ± 0.96	<LOQ
Sausage with walnut	Walnuts	N.D.	N.D.
Chocolate	Almond and Hazelnut traces	37.99 ± 0.33	<LOQ
Chocolate Bar I	Tree nut traces	32.42 ± 0.16	2.5
Chocolate Bar II	Not declared	31.32 ± 0.21	5.7
Cookies with fiber	Not declared	31.02 ± 0.30	7.1
Cookies with chocolate	Not declared	32.25 ± 0.46	5.7

* Estimated quantity of peanut by substitution of the obtained mean Ct in the standard curve from mixtures spiked with untreated peanut.

## Supplementary Materials

The following supplementary material is available online at https://www.mdpi.com/article/10.3390/foods10061421/s1, Table S1: Preparation of binary mixtures of peanut in spelt wheat flours, Table S2: Primers used for sequencing purposes; Table S3: Detection of mat K target by probe-based real-time PCR in untreated (control) and treated spiked samples; Figure S1: Sequence alignment of two clones of partial Ara h 6-allergen coding gene. Figure S2: flow chart summarizing protocols, procedures, markers, and the main findings of this study.
