# Supplementary material for: Detection of Peanut Allergen by Real-Time PCR: Looking for a Suitable Detection Marker as Affected by Processing
Source: Foods. 2021 Jun 18;10(6):1421. doi: 10.3390/foods10061421 (PMC8234062; doi:10.3390/foods10061421)
Supplement: Supplementary file 1 [file foods-10-01421-s001.zip › foods-1201722-supplementary.pdf]

**Table S1.** Preparation of binary mixtures of peanut in spelt wheat flours.

| Mixture | % | mg/kg |
|---|---|---|
| **S1** | 10 | 100,000 |
| **S2** | 1 | 10,000 |
| **S3** | 0.1 | 1,000 |
| **S4** | 0.05 | 500 |
| **S5** | 0.01 | 100 |
| **S6** | 0.005 | 50 |
| **S7** | 0.001 | 10 |
| **S8** | 0.0001 | 1 |
| **S9** | 0.00005 | 0.5 |
| **S10** | 0.00001 | 0.1 |

**Table S2**. Primers used for sequencing purposes.

| Oligo | Sequence 5′→ 3′ | Amplicon |
|---|---|---|
| **trnH-psbA fw** | ACATCCGCCCAAAGGAGAAAT | 414 |
| **trnH-psbA rev** | TCTGGTTTACCGCGTTAGGT | |
| **rpl 16 fw** | GCGATGGGAACGACGAAAAC | 493 |
| **rpl 16 rev** | ACGGCTCCTCGCGAATAAAA | |
| **mat k fw** | TGGACTCGCCTCTGGTCAT | 392 |
| **mat k rev** | CCAGATGGATAGGATAGGGTATTCG | |
| **Ara h 6 fw** | AGTACTCGATCCTCCGACCA | 392 |
| **Ara h rev** | AAGCCATAAGAGCACACCGAA | |

**Table S3**. **Detection of mat K target by probe-based real-time PCR in untreated (control) and treated spiked samples.** DNA isolation protocol was DNeasy Plant Pro Kit (Qiagen, Protocol 1) for all samples.

| Peanut quantity (mg/kg) | Control[1] | Boiling 60 min | DIC 7b 120s |
|---|---|---|---|
| 100000 | 17.55 ± 0.17 | 18.69 ± 0.25 [ns] | 24.15 ± 0.29 |
| 10000 | 21.52 ± 0.30 | 23.30 ± 0.30[ns] | 28.27 ± 0.62 |
| 1000 | 24.17 ± 0.17 | 26.15 ± 0.28 | 31.51 ± 1.14 |
| 100 | 27.89 ± 0.14 | 28.33 ± 0.25 [ns] | 34.62 ± 0.68 |
| 10 | 30.77 ± 0.25 | 33.36 ± 0.29 | 38.56 ± 0.33† |
| 1 | 33.22 ± 0.20 | 34.17 ± 0.75 | 39.69 ± 0.25 (50%)† |
| 0.5 | 32.74 ± 0.15† | 36.47 ± 0.45 | N.A. |
| 0.1 | 33.87 ± 0.58† | N.A. | N.A. |
| Slope | -3.14 | -3.17 | -3.46 |
| Efficiency (%) | 108.30 | 106.73 | 94.43 |
| $R^2$ | 0.995 | 0.982 | 0.995 |

| Peanut quantity (mg/kg) | AU121⁰C 15 min | AU121⁰C 30 min | AU138⁰C 15 min | AU138⁰C 30 min |
|---|---|---|---|---|
| 100000 | 20.93 ± 1.14 | 25.01 ± 0.28 | 29.50 ± 0.13 | 38.68 ± 0.89 (50%) |
| 10000 | 24.84 ±1.20 | 29.55 ± 0.33 | 30.63 ± 0.05 | 38.92 ± 0.72 (50%) |
| 1000 | 28.23 ± 0.67 | 33.28 ± 0.29 | 35.54 ± 0.06 | 39.41 ± 0.40 (50%) |
| 100 | 30.87 ± 0.78 | 35.39 ± 0.43 | 37.09 ± 0.33† | 39.70 ± 0.34 (25%) |
| 10 | 32.75 ± 0.21† | 37.93 ± 0.49† | 39.6 ± 0.32 (75%)† | N.D. |
| 1 | 34.79 ± 1.01† | 39.87 ± 0.15† (25%) | N.D. | N.D. |
| Slope | -3.32 | -3.48 | -3.02 | -- |
| Efficiency (%) | 100.05 | 93.65 | 114.23 | -- |
| $R^2$ | 0.993 | 0.975 | 0.885 | -- |

[1]Ct±SE

[2]Percentage of positive amplification

N.A. Not assayed

N.D. Signal was not detected after 40 cycles of amplification

†Detection is possible but Ct is not in the calibration curve.

[ns] Not significant differences in mean Ct values compared to untreated control (t-student, p >0.05).

```
1     AGTACTCGATCCTCCGACCAGCAACAG-AGGTGCTGCGATGAGCTGAACGAGATGGAGAA  59
      ||||||||||||||||||||||||||| |||||||||||||||||| || ||||||||||
1     AGTACTCGATCCTCCGACCAGCAACAGCAGGTGCTGCGATGAGCTGGACCAGATGGAGAA  60

60    CACACAGAGATGCATGTGCGAGGCATTGCAGCAGATAATGGAGAACCAGTGCGATAGGTT  119
      |||| |||||||||||||||||||||||||||||||||||||||||||||||||||||||
61    CACAGAGAGATGCATGTGCGAGGCATTGCAGCAGATAATGGAGAACCAGTGCGATAGGTT  120

120   GCAGGACAGGCAAATGGTGCAGCAGTTCAAGAGAGAGCTCATGAACTTGCCCCAACAGTG  179
      ||||||||||||||||||||||||||||||||| ||||||||||||||||| ||||||||
121   GCAGGACAGGCAAATGGTGCAGCAGTTCAAGAGGGAGCTCATGAACTTGCCTCAACAGTG  180

180   TAACTTTAGGGCACCACAGCGTTGCGATTTGGACGTGAGTGGCGGCAGATGCTAGACTCA  239
      |||||| |||||||||||||||||||||||||||||||||||||||||||||||||||||
181   TAACTTCAGGGCACCACAGCGTTGCGATTTGGACGTGAGTGGCGGCAGATGCTAGACTCA  240

240   AAAATAATAATCTGTGCCAAAACAAACTAGTAGGAAGTAGCTTATGAGCTATTATGTATG  299
      |||||||||||||||||||||| |||||||||||||||||||||||||||||||||||||
241   AAAATAATAATCTGTGCCAAAAGAAACTAGTAGGAAGTAGCTTATGAGCTATTATGTATG  300

300   CTTGTTTCGTTAATAATAAACATCATCACTGTATGAATGTGGTGATAGCTAGGTAAGGTT  359
      |||||||||||||||||||| ||||||||||||||||||||||||    |||||||||||
301   CTTGTTTCGTTAATAATAAATATCATCACTGTATGAATGTGGTGA----TAGGTAAGGTT  356

360   ATATGAGCACCTTCGGTGTGCTCTTATGGCTT  391
      ||||||||||||||||||||||||||||||||
357   ATATGAGCACCTTCGGTGTGCTCTTATGGCTT  388
```

**Figure S1. Sequence alignment of two clones of partial Ara h 6-allergen coding gene.** Primers and probe designed for real-time PCR experiment are squared in red and green respectively.

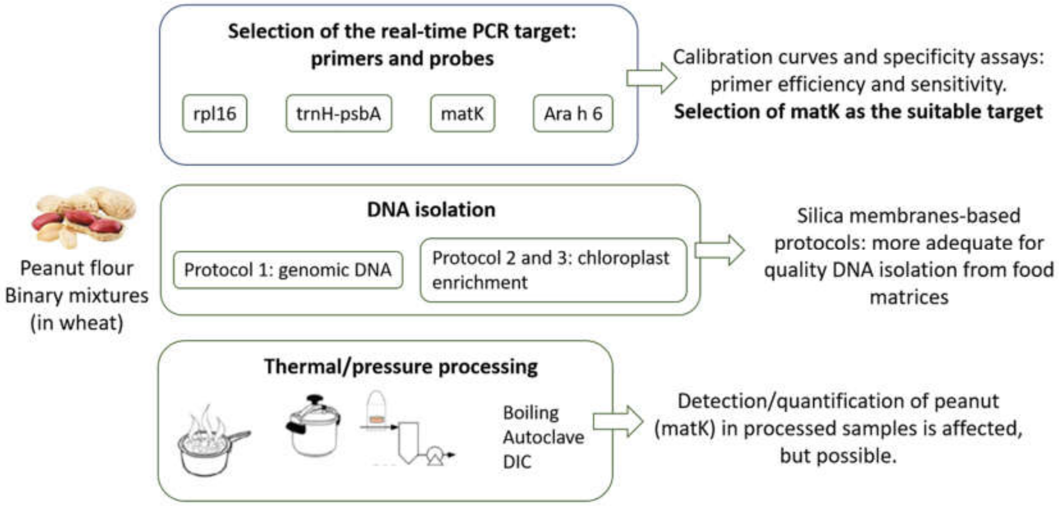


**Figure S2.** Workflow summarizing protocols, procedures, markers and the main findings of this study.
